# Reflections on the scope and the future of Particle and Fibre Toxicology

**DOI:** 10.1186/1743-8977-8-13

**Published:** 2011-04-01

**Authors:** Flemming R Cassee

**Affiliations:** 1 Centre for Environmental Health, National Institute for Public Health and the Environment, P.O. box I, 3720 BA BILTHOVEN, The Netherlands

## Abstract

Within 5 years of its first publication in December 2004, *Particle and Fibre Toxicology *has become a well recognized open access, peer-reviewed, online journal with an (unofficial) impact factor of 5.5. This major achievement is due to the dedication of former Editors-in-Chief Professors Ken Donaldson and Paul Borm, and, of course also due to the high quality of manuscripts that have been submitted by authors from all over the world. Recent years have shown a significant increase in papers dealing with nanomaterials and nanotoxicology, whilst the small margin between ambient PM exposure and current standards continues to provide a constant flow of manuscripts on this topic. This however, does not imply that we can relax now.

## Achievements

To date, *PFT *has received over 270 manuscripts, with a sharp increase in the number of submissions in 2010 compared to the previous year. Of the 270 manuscripts submitted, approximately 50% have been accepted for publication and this percentage is likely to decrease due to a more stringent editorial policy, resulting in the publication of even higher quality papers in the future. Thirty percent of all the publications were from North-America, 63% from Europe and the remaining portion were from Asia and Australia. The number of papers that deal with engineered nanoparticles versus those that cover ambient PM remains similar. However, very few papers focus on workplace or indoor exposure. Most important is the fact that the average number of citations per paper is still increasing, which of course will strongly affect the journal's impact factor.

Several toxicological papers have been rejected due to a lack of exposure characterization. Although it has been acknowledged that particle characterization is often an expensive and laborious part of a study, it is crucial for the interpretation of the study results, particularly in the area of nanomaterials. As such, (associate) editors and reviewers of *PFT *will critically evaluate the soundness of exposure characterization in submitted papers. Primary particle size is often very different from what we see when particles interact with biological systems. This can be due to particle agglomeration or aggregation during the production, handling and storage of particles. Another major influence of particle size is the coating of the particle, whether or not on purpose. Protein coronas and other particle surface modifications have been described in several studies as having major impacts in the toxic properties of nanomaterials and particles [1][2]. Despite the exponential increase in the number of publications dealing with toxic properties of nanomaterials, appropriate data depicting health and environmental risk is deficient and consequently inadequate for carrying out thorough risk assessment on their use [3]. This is mainly due to the fact that toxicological studies that have applied realistic exposure scenarios (including relevant conditions) as well as the techniques to discriminate engineered from unintentionally produced particles and fibres are complicated or not easy to apply in real world situations.

## Future directions

Research articles should always contain a good description of the particles in the formulation in which they have been studied, either in suspension for use in *in vitro *systems, oral or dermal applications, intravenous injections, or as aerosols for inhalation studies. This demands closer collaboration with physicist, chemists and experts on exposure assessment and as such implies multidisciplinary studies. Inclusion of appropriate control/benchmark particles is always encouraged so that the particle effects can be contextualised. Studies that have been performed under well-defined conditions and preferably with exposure scenarios that are within range of real life exposure are particularly welcome for publication in *PFT*. *PFT *functions as a forum for scientific debate and communication between toxicology and disciplines that develop particles and fibres as well as those that assess the risk for adverse human health and environmental effects, including epidemiology, material sciences and nanomedicine. *PFT *will remain an open access, peer-reviewed, online journal for novel scientific data, hypotheses and reviews on the toxicological effects. Moreover, since risk assessment of particles and fibres requires information on the hazard as well as the exposure, submission of research into exposure assessment is encouraged, provided that links are being made to toxicology [4]. *PFT *also welcomes studies that are explicitly done for the purpose of risk assessment, e.g. those following OECD Technical Guidelines.
